# Radiopaque Polyurethanes Containing Barium Sulfate: A Survey on Thermal, Rheological, Physical, and Structural Properties

**DOI:** 10.3390/polym16213086

**Published:** 2024-10-31

**Authors:** Heitor Luiz Ornaghi Júnior, Benoit Duchemin, Sanae Azzaye, Márcio Ronaldo Farias Soares, Bárbara Schneider, Carlos Henrique Romoaldo

**Affiliations:** 1 Mantova Industria de Tubos Flexíveis, R. Isidoro Fadanelli, 194, Centenário, Caxias do Sul CEP 95045137, Brazil; mfsoares@gmail.com (M.R.F.S.); bscheider.89@gmail.com (B.S.); projeto@mantova.ind.br (C.H.R.); 2 Laboratoire Ondes et Milieux Complexes (LOMC), UMR 6294, CNRS-Université le Havre Normandie, 76600 Le Havre, France; benoit.duchemin@univ-lehavre.fr (B.D.); sanae.azzaye@univ-lehavre.fr (S.A.)

**Keywords:** radiopacity, polyurethane, barium sulfate, biomedical applications

## Abstract

Radiopaque polyurethanes are extensively used in biomedical fields owing to their favorable balance of properties. This research aims to investigate the influence of particle concentration on various properties, including rheological, radiopacity, structural, thermal, and mechanical attributes, with a thorough analysis. The findings are benchmarked against a commercial product (PL 8500 A) that contains 10% weight barium sulfate. Two more thermoplastic polyurethanes (TPU) were formulated with two different concentrations of barium sulfate (10 wt.% and 20 wt.%) and compared to the commercially available product. FTIR demonstrated similar absorption bands among all samples, indicating that the fabrication method did not impact the TPU matrix. DSC indicated a predominantly amorphous structure for PL 8500 A compared to the other samples, while the kinetic degradation was more influenced by the higher barium sulfate content. The rheological analysis showed a decrease in the complex viscosity and storage modulus with the radiopacifier and an increase in the radiopacity, as demonstrated by the X-radiography. X-ray microtomography showed a more spherical particle format with a heterogeneous particle structure for PL 8500 A compared to the other polyurethanes. These findings enhance the comprehension of the structure–property relationships inherent in these materials and facilitate the development of customized materials for targeted applications.

## 1. Introduction

**Figure 1:**
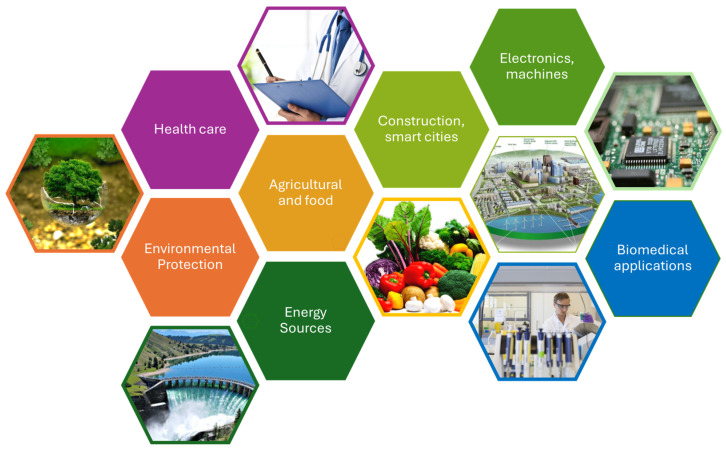
Different applications of nanoparticles and nanomaterials in biomedical products. The figure is based on [12].

The use of biomaterials to support, enhance or replace damaged tissue or a biological function in medical applications has been significantly increasing around the world [1,2,3]. Artificial joints, ligaments, heart valves, and dental implants are some examples of biomaterials successfully applied into the human body [4,5,6]. To enhance the performance of biomaterials, nanoparticles have been used to promote positive effects on processing, radiopacity, cell adhesion, viability, and migration, in the majority of studies [7]. Biomaterials can be classified, as follows, by: type (hybrid, organic, inorganic, carbon); fabrication (spinning, hydrothermal, 3D printing); risk and toxicities (eye contact, digestion, skin penetration); and unique properties (depending on the size composition and shape functionality) [8]. Due to the wide variety of nanoparticles and nanostructures, these materials can be applied via drug delivery, bioimaging, diagnosis, and antimicrobials, among others, as demonstrated in Figure 1 [9,10,11].

Considering medical implants, it is highly recommended that the medical procedure be non-invasive and easily detected inside the human body. Some of the techniques used for this purpose are tomography and radiography. It is noteworthy to mention that most polymers are not intrinsically radiopaque, which makes visualization in the human body difficult. The reason is that most polymers are composed of atoms with low electron density and low specific gravity, such as carbon, hydrogen, and nitrogen; hence, the resulting image is radiolucent (transparent to X-rays or other forms of radiation) [13]. To improve the radiopacity of polymers, the average electron density and the specific gravity of the atoms must also be increased by incorporating heavier atoms (iodine or barium) in the polymer backbone through synthesis or by adding ceramic/metallic particles into the polymeric matrix [13]. An alternative is to mix nanoparticles (such as barium sulfate) with the polymer (such as polyurethane) in the process and obtain the radiopaque material.

Thermoplastic polyurethanes (TPUs) are one of the most versatile polymers due to the potential for producing tailor-made properties for specific applications; these can be found in many biomedical applications, including heart valves, mammary implants, suture materials, and others [14]. All commercially available polyurethanes can contain particles/nanoparticles such as barium sulfate, tungsten, and bismuth salts that have adequate properties, radiopacity, biocompatibility, and so on. To date, many studies have been conducted regarding the fabrication and production of radiopaque TPU or TPU blends. Sang et al. [15] developed drug-loaded radiopaque iodinated poly (lactic acid)-polyurethane beads for chemoembolization therapy. The materials showed sufficient radiopacity, in vitro non-cytotoxicity with human adipose-derived stem cells, and in vivo biocompatibility and degradability in a rabbit model via intramuscular implantation. Dawlee and Jayabalan [16] developed an intrinsically radiopaque and nontoxic polyurethane with an iodinated chain extender, 4,4′-isopropylidenebis [2-(2,6-diiodophenoxy)ethanol]. The material has cytocompatibility with L929 mouse fibroblast cells and has reasonable tissue compatibility, even after 12 weeks of implantation. James et al. [13] added a 5-iodine-containing molecule, N-(2,6-diiodocarboxyphenyl)-3,4,5-triiodobenzamide, onto the polymer backbone of a medical-grade aliphatic polyurethane, Tecoflex^®^ 80A. The authors claimed an equivalent radiopacity to that of a 2 mm-thick aluminum wedge by incorporating about 8% iodine in the polymer backbone. The modified polyurethane showed a lower thermal stability due to the reduced extent of intermolecular hydrogen bonding among the hard segments of the end product compared to the unmodified polyurethane. Kiran et al. [17] iodinated a bisphenol-A and used it as a chain extender for the preparation of a radiopaque PU. The final material was shown to be highly radiopaque, non-cytotoxic when tested in vitro using L929 mouse fibroblast cells, and had the potential to be synthesized with desirable properties for different applications. Kashyap et al. [18] developed a radiopaque, porous shape memory polyurethane for endovascular embolization. The material has a high volumetric expansion and enhanced biological activity, which is ideal for minimally invasive surgical procedures. Lu et al. [19] analyzed the rheology and processing of polyurethane filled with barium sulfate. The rheological properties showed a decrease in the shear viscosity and elasticity, in comparison with the neat PU, between 190 and 200 °C. In addition, hydrolysis occurred in the polymer due to the humidity in the barium sulfate, which decreased the molecular weight of the polyurethane.

The final properties of radiopaque polyurethanes depend on several variables such as synthesis conditions, polyol/isocyanate ratio, the type of chain extender, the amount and shape/format of the filler incorporated, and so on. All these variables will affect the chain morphology, network structure, chain dynamics, and, consequently, the final properties of the polyurethane [13,16,18,19]. For example, the higher the amount of barium sulfate incorporated in the TPU, the lower the viscosity and processing temperature, and better flow stability is achieved. Other properties, such as thermal stability, cytotoxicity, and mechanical properties, can also be modified by the incorporation of barium sulfate or other particles. As demonstrated earlier, many studies can be found on obtaining radiopaque polymers using ceramic/metallic particles or by introducing heavy atoms (barium or iodine) in the synthesis of the polyurethane as part of the polymer backbone. However, the incorporation of barium sulfate as a masterbatch in polyurethane is very promising, mainly for industrial purposes such as injection and extrusion.

This study aimed to evaluate the effect of particle concentration on rheological, radiopacity, structural, thermal, and mechanical properties, which were analyzed in detail. The results were compared with one commercial-grade polyurethane (PL 8500 A) containing 10% wt. of barium sulfate, named PL 8500 A-10%. TPU were processed (using a masterbatch procedure) with two distinct barium sulfate contents (10 wt.% and 20 wt.%), named TPU10% and TPU20%, respectively. The effects of the nanoparticles on TPU from the micro to the macro scale were discussed in detail.

## 2. Materials and Methods

### 2.1. Materials

The following two types of polyurethanes were used: TPU PL 8500 A containing 10 wt.% barium sulfate (grade TPU 85 ether) (grade 26379-23-5) (HididaPlast Indústria de Plástico Ltda, São Paulo, Brazil); and TPU Wanthane^®^ WHT-1190B (grade 9018-04-06) (Wanhua Chemical Plastics, Yantai, China). For the TPU Wanthane^®^ (ester), 10 wt.% and 20 wt.% of barium sulfate from Univar Solutions (Redmond, WA, USA) were incorporated. The incorporation was performed by an external company, Supercor Indústria de Pigmentos Plásticos (Caxias do Sul, RS, Brazil), and was received in pellet format. For all tests, the pellets were used. For the radiopacity and microtomography rectangular bars were injected in a mini-injector at 8 bar pressure at 200 °C (Homemade fabrication, Caxias do Sul, Brazil). Four different samples were analyzed, as follows: PL 8500 A-10%; TPU; TPU-10%; and TPU20%. With the exception of PL 8500 A-10%, the remaining polyurethanes were produced using materials from Wanhua.

### 2.2. Methods

#### 2.2.1. Particle Characteristics

The barium sulfate was obtained from Univar Solutions with the following characteristics: particle size (0.7 μm); purity (min. 99%); mesh residue 325 (max. 0.02%); pH (6.5–9.5); whiteness (min. 97%); water solubility (max. 0.15%); and oil absorption (15–18%).

#### 2.2.2. Fourier Transform Infrared (FTIR) Spectroscopy

The nanocomposites (pellets) were evaluated by Fourier transform infrared spectroscopy (FTIR) on a Perkin Elmer Impact 400 spectrometer (PerkinElmer Inc., São Paulo, Brazil) (4000−400 cm^−1^ region, 32 scans, and 4 cm^−1^ resolution) using the attenuated total reflectance mode (ATR, diamond at 45°).

#### 2.2.3. Thermal Behavior

The DSC was performed using DSC Q2000 (Waters/TA Instruments, New Castle, DE, USA), aiming to evaluate changes in the melting, crystallization, and glass transition temperature of the polyurethanes. The first heating rate was performed from 20 to 250 °C, followed by a cooling ramp until −50 °C and by a second heating rate until 250 °C. Only the cooling and second heating curves were evaluated. The first curve was conducted to eliminate the thermal and processing effects of the TPUs.

Thermogravimetric analysis (TGA) was performed using a TGAQ500 (Waters/TA Instruments, New Castle, DE, USA). The thermal degradation study was conducted at five distinct heating rates, as follows: 2 °C·min^−1^; 5 °C·min^−1^; 10 °C·min^−1^; 20 °C·min^−1^; and 40 °C·min^−1^. The thermal kinetic study was performed according to ICTAC recommendations [20] using free open-source thermo-kinetic software (THINKS) (version 2016-2020) [21]. More details about the kinetic procedure can be found in the Supplementary Materials (S1).

#### 2.2.4. Rheological Tests

The rheological tests were performed using an Anton Paar Rheoplus MCR 301 (Anton Paar Instruments, São Paulo, Brazil). The shear rate range measured was from 1 s^−1^ to 1000 s^−1^. The gap between the plates was 1 mm in height at a temperature of 200 °C, using 5% of the strain amplitude.

#### 2.2.5. X-Radiography

The radiopacity was measured in a POSKOM machine, model PXP-20HF PLUS. The X-ray plate was the DR Venu model 1717X. Both were obtained from Alumed Equipamentos Veterinários (Campo Belo, SP, Brazil). The X-radiograph was performed in the Salute Clinica e Oncologia Veterinária (Caxias do Sul, RS, Brazil).

#### 2.2.6. X-Ray Microtomography

X-ray microtomography was conducted on an easytom150 from RX solutions (Annecy, France) equipped with a 150 kV microfocus source with a tungsten target, a 7 μm focal spot size, and a 200 μm-thick beryllium window. Reconstructions were conducted with proprietary Xact v.1.1 software and resulted in an image stack with a voxel size of 20.0 μm. The image treatment was performed with FIJI software (available on https://imagej.net/software/fiji/, (accessed on 28 October 2024)) using the 3D object counter library to produce particle size and surface estimations.

## 3. Results and Discussion

### 3.1. Fourier Transformed Infrared (FTIR) Spectroscopy

**Figure 2:**
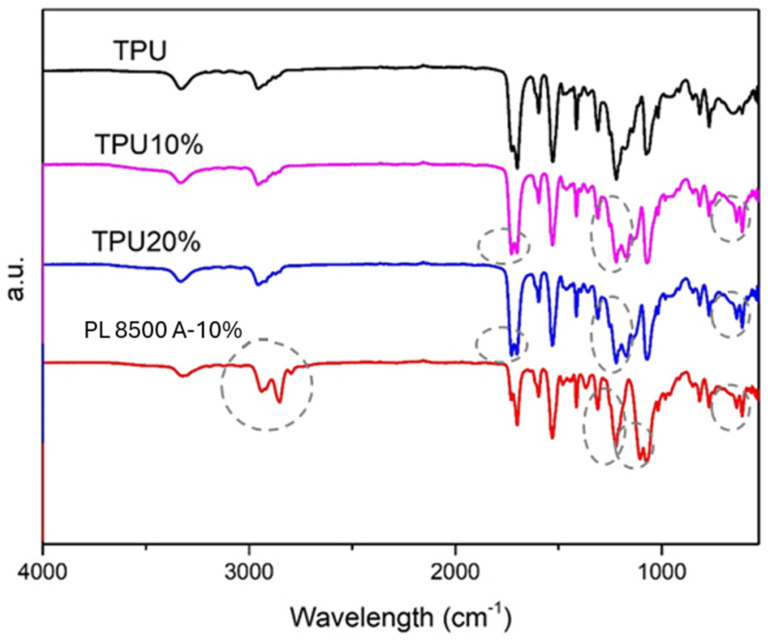
FTIR spectra of PU sample and the nanocomposites. The gray circles highlight the regions with higher differences in the spectra.

The FTIR spectra of the TPU and the nanocomposites are presented in Figure 2.

The TPU absorption bands are described as follows. The C=O stretching of the ester carbonyl group can be observed at 1765–1637 cm^−1^ and can be attributed to polyol. At wavelengths of 1463 cm^−1^, 2922 cm^−1^, and 2855 cm^−1^, the symmetric and asymmetric stretching vibrations of the -CH_2_ group can be observed [22]. At 722 cm^−1^, the rocking vibration of -CH_2_ can be seen. Two distinct absorption bands (3010 cm^−1^ and 1640 cm^−1^) are attributed to the double carbon bond (C=C). The stretching of C-N and N-H is observed at 1525 cm^−1^. The stretching vibration of the -NH of the urethane group is observed at 1600 cm^−1^. The stretching of the (C-C(=O)-O) (at 1217 cm^−1^ and 955 cm^−1^) and the stretching of the CH_2_ and CH_3_ (1307 cm^−1^ and 1500 cm^−1^) are also observed [23]. The gray dotted circles represent the main changes observed in the absorption peaks of the spectra by the incorporation of barium sulfate. The appearance of two bands in the 608–640 cm^−1^ region is observed by BaSO_4_ incorporation due to the out-of-plane bending vibration of the SO_4_^2-^. Additionally, S-O stretching is found in the region of 1179–1083 cm^−1^. The bands at the absorption range of 1192–1072 cm^−1^ are attributed to the symmetrical vibration of SO_4_^2-^. Finally, the absorption bands at 2855 cm^−1^ and 2925 cm^−1^ are due to the symmetric and asymmetric vibrations of -CH_2_ and -CH_3_ [24,25]. Comparing all spectra with the PL 8500 A-10%, similarities can be noted (the ether spectra are very similar to the ester spectra), with slight differences in some band intensities.

### 3.2. Thermal Behavior

**Figure 3:**
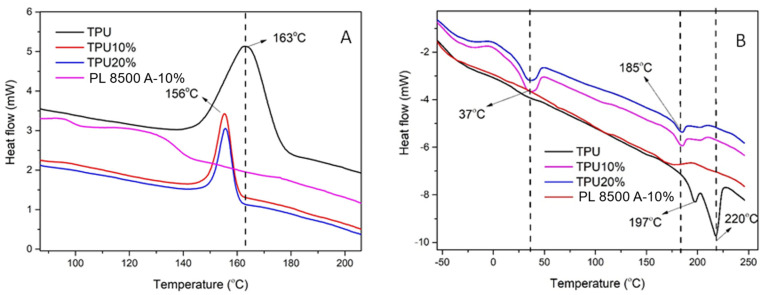
DSC curves of PU sample and the nanocomposites: (**A**) cooling ramp; and (**B**) second heating rate.

The DSC curves are presented in Figure 3A,B for the TPU and all nanocomposites. The cooling ramp (Figure 3A) and the second heating ramp (Figure 3B) were compared, aiming to verify changes in the crystallization (Tc) and melting (Tm) temperatures with barium sulfate incorporation. There are several factors that affect crystallization such as polymer molecular weight, chemical structure, processing conditions, additives, etc. Briefly, if the polymer cannot rearrange in a regular and symmetrical pattern (crystal), no crystallization is observed [26]. Almost all polymers are amorphous in the molten state (with the exception of polymers with a nematic–isotropic transition) with a high entropic factor [27,28]. By cooling, both the entropy and the polymeric chain mobility also decrease, and the energy that has not been dissipated is used to form crystals (considering crystallizable polymers) that are visualized as a peak in the DSC curve. Hence, if no peak is observed, no crystals are formed. Considering Figure 3A, the crystallization curves of TPU occurred at Tc = 163 °C in a wide temperature range (140–180 °C). With the incorporation of barium sulfate (TPU10% and TPU20%), the curves were identical, with a maximum at Tc = 156 °C distributed in a narrower temperature range of 150–160 °C. For PL 8500 A-10%, no peak was observed, indicating no existence of crystalline portions or the existence of a small quantity of crystals not detected by the analysis. In this case, the material can be considered amorphous [29,30].

In the second heating ramp (Figure 3B), the maximum temperature was found at Tm = 220 °C with a small shoulder at Tm = 197 °C. With the incorporation of barium sulfate (TPU10% and TPU20%), the existence of two peaks at Tm = 37 °C and Tm = 185 °C is noted, i.e., the material crystallizes earlier at a shorter interval range. If the material is used in an injection molding process, lower injection temperatures and a faster production of the parts are attained. The results indicate that the barium sulfate particles can act as crystallization points (for TPU10% and TPU20%), leading to a homogenous crystallization structure (observed by the narrower and lower area of the Tm curves). The melting temperature at Tm = 37 °C found for TPU10% and TPU20% is due to the masterbatch formulation from Supercor Company (probably due to the addition of plasticizer or an additive).

**Figure 4:**
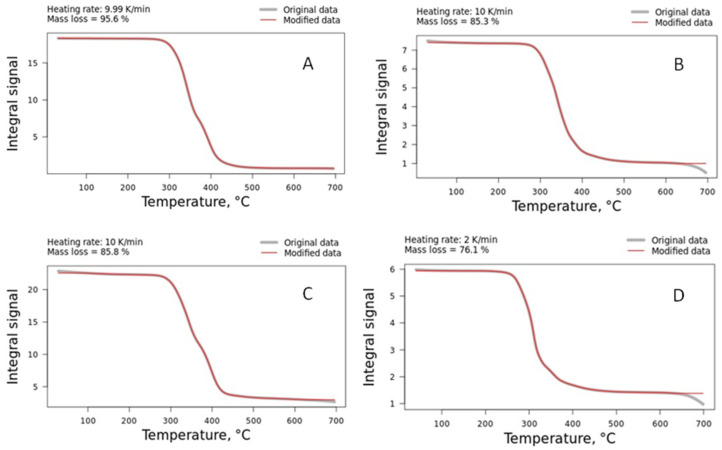
Comparative curves for: (**A**) TPU; (**B**) TPU10%; (**C**) PL 8500 A-10%; and (**D**) TPU20% at some selected heating rates.

The thermal degradation behavior of the TPU and the composites is presented in Figure 4. Similar behavior was found for all materials, i.e., a plateau that extended from 20 to 300 °C, followed by a rapid curve decreasing at a shorter time interval, followed by a terminal plateau. In most cases, the main degradation event showed two distinct peaks. In the case where one peak was observed, we attributed this to the structural heterogeneity of the TPU or the mixture (TPU/barium sulfate). The polyurethane has hard and soft segments that degrade at different stages; the degradation of the hard segments, where more co-products are generated, is the first stage. The scission of hard segments produces different chemicals, such as isocyanate, alcohol, olefin, carbon dioxide, and primary and secondary amines, while the soft chemicals degrade into smaller fragments [31]. This is the reason why there is a considerable variation in the terminal plateau for the samples (a sum of the processing plasticizer of the TPU, barium sulfate concentration, and masterbatch additives).

**Figure 5:**
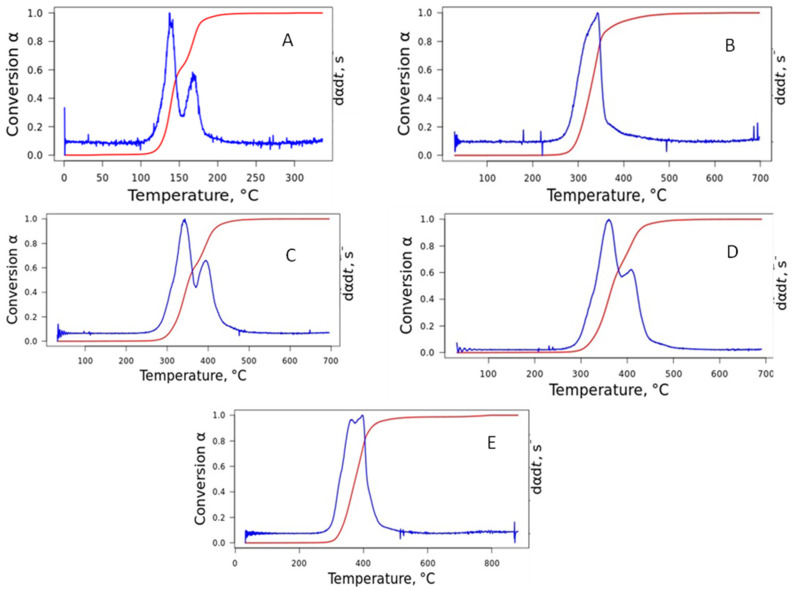
Conversion rate of the TPU at different heating rates: (**A**) 2 °C·min^−1^; (**B**) 5 °C·min^−1^; (**C**) 10 °C·min^−1^; (**D**) 20 °C·min^−1^; and (**E**) 40 °C·min^−1^.

Figure 5A–E represents the conversion rate as a function of temperature for TPU at different heating rates. In general, two distinct peaks are observed for all heating rates. It is observed in a sigmoidal format (red curve), indicating an autocatalytic reaction model in two stages, which is similar to that seen in the literature [32]. Kolmogorov–Johnson–Mehl–Avrami and Sestak–Berggren are two examples of sigmoidal models. It was considered that the processes at the initial and final stages demonstrate accelerating and decelerating behaviors, with the process rate reaching its maximum at some intermediate value. It was also noted that the noisy signal has no significant influence on the heating rate.

**Figure 6:**
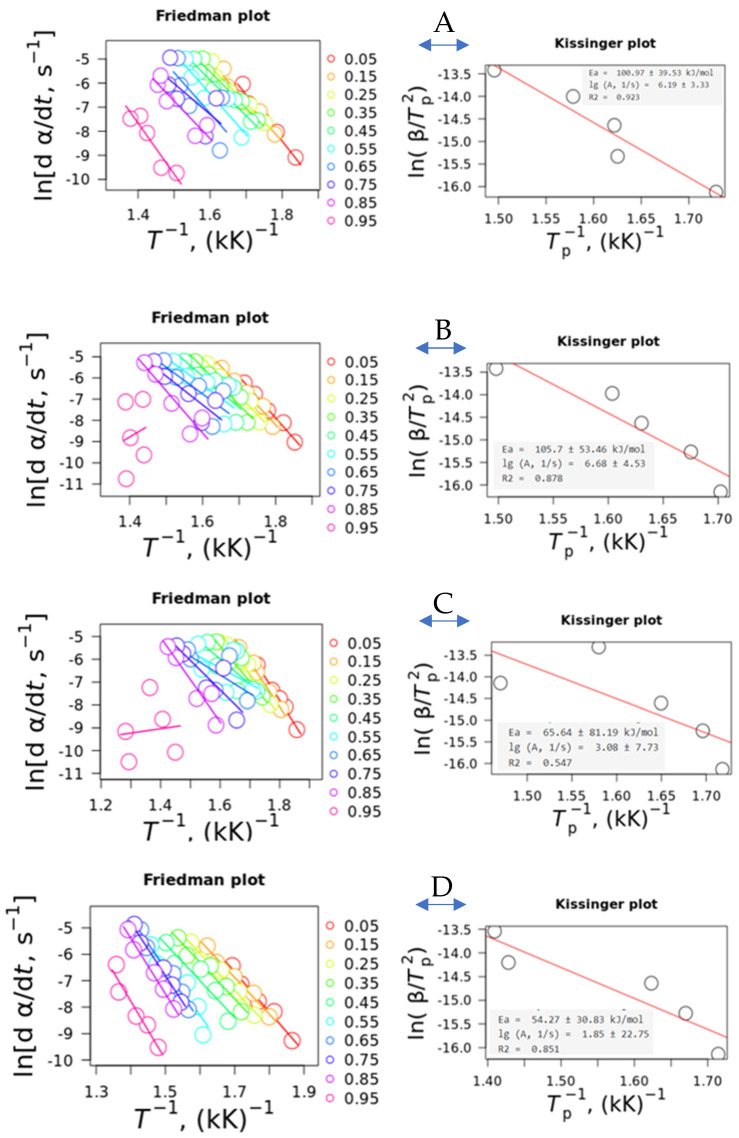
Friedman and Kissinger plots for: (**A**) TPU; (**B**) TPU10%; (**C**) TPU20%; and (**D**) PL 8500 A-10%, respectively.

The Friedman and Kissinger plots are presented in Figure 6. Regarding the Friedman plots, it can be seen that the slope of the curves from α = 0.05–0.85 is similar for the TPU and the nanocomposites, with the exception of the TPU20%, where in α = 0.45, the slope is more accentuated, indicating higher activation energy. Additionally, at α > 0.85 for TPU10% and TPU20%, a poor fit can be observed, which is typical for most polymeric materials due to the end of the pyrolytic process that does not fit according to the theoretical model [20,33]. In the case of PL 8500 A-10%, similar behavior for all α were observed in a wide temperature range compared to the other TPUs. Additionally, for α < 0.05 or 0.1, due to the initiation period of pyrolysis, the kinetic behavior can usually be neglected [33]. The Kissinger plots indicated that all *E_a_* and *A* can be considered as similar when considering the high standard error values. This error can be explained because Kissinger considers only the main peak, and in some cases the presence of more than one peak is observed, leading to the discrepancy in the results. In our case, the use of Kissinger’s method is shown to be inefficient, mainly when it is considered that the peak showing at approximately α = 0.5 has a high standard error associated with it. Furthermore, the Kissinger plot can be better used if the activation energy with the conversion degree is the same (or very similar) at the early or late portions of the reactions [20,34,35]. Another problem pointed out by Vyazovkin [35] is that the model is not accurate for reactions that do not follow first-order reactions such as diffusion or random scission. In addition, it cannot be ignored that degradation can be a multi-step process, in spite of some linearity visualized in the activation energy vs. the conversion degree. However, considering the standard error associated, it is difficult to point out if the process follows a single- or multi-step mechanism.

**Figure 7:**
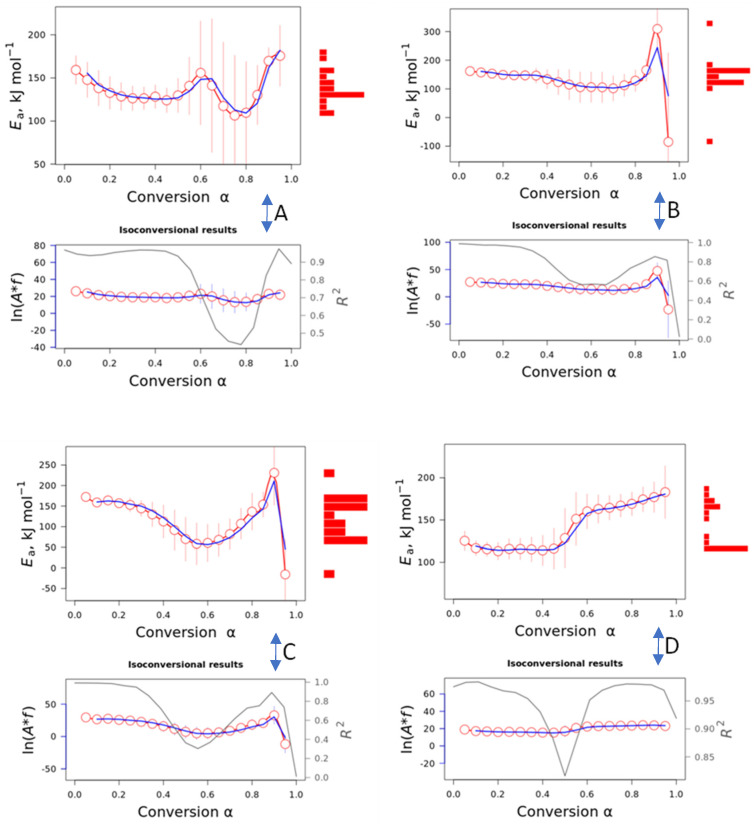
Activation energy and preexponential factor dependency with the conversion degree for: (**A**) TPU; (**B**) TPU10%; (**C**) TPU20%; and (**D**) PL 8500 A-10%. The activation energy and preexponential factor plots represent the Friedman (red circle color) and Vyazovkin (blue line color) mathematical treatment. The gray line color of preexponential plot represent the R^2^ while the red bar column in the activation energy factor represent the activation energy values concentration.

Figure 7A–D shows the activation energy and pre-exponential factor dependency with the conversion degree for the TPU and the nanocomposites.

For TPU, TPU10%, and TPU20%, similar initial *E_a_* values (~160 kJ·mol^−1^) can be observed; however, for TPU, a slight decrease is observed until α = 0.5, followed by an increase and a further decrease, showing no linearity in the *E_a_* values with the conversion. With the incorporation of 10% barium sulfate (TPU10%), the *E_a_* curve showed a more linear dependency with α, which indicates a homogeneous degradation process. Upon further incorporation of barium sulfate (20%), the *E_a_* slightly decreases until a minimum of 50 kJ·mol^−1^, which indicates that the barium sulfate decreases the activation energy but does not necessarily interfere with the thermal stability of the nanocomposites. Phenomenologically, if the thermal stability of the two materials (polyurethane and barium sulfate) is different, the heating transfer also differs. In some cases, the degradation of one component can autocatalyze the other component; however, in other cases this does not occur. Sometimes this process can be easily detected by differences in the activation energy values vs. the conversion degree. For PL 8500 A-10%, the opposite effect is observed compared to TPU20%, i.e., a lower initial *E_a_* is observed at 110 kJ·mol^−1^ and increases to 150 kJ·mol^−1^ for α = 0.5. All pre-exponential factors showed linearity with the conversion degree and similar results for all TPUs and nanocomposites (20 s^−1^). The R^2^ values were inconsistent, mainly from α = 0.5, indicating that as the reaction proceeds, the error is very high, mainly in the region where two peaks were observed in the DTG curve. According to Vyazovkin [36], a more complete understanding of the kinetic process is necessary to determine the preexponential factor as well as the activation energy dependency as a function of the conversion degree. Usually, a higher thermal stability can be attributed to a higher energy, not alone, but associated with the preexponential factor behavior. The higher thermal stability of the samples indicates an increase in the activation energy values characteristic of a deceleration of the process. If a higher energetic barrier is required for an increase in the kinetic energy of the reactant molecule, this phenomenon is accompanied by an increase in the temperature. An increase in the *E_a_* without a change in *A* is indicative of a deceleration process caused by shifting to a higher temperature. Additionally, there is a deceleration when *E_a_* decreases with *A*. As will be further shown, the most probable reaction mechanism is diffusion, which is a deceleration-type mechanism [20]. The main problem visualized in our samples is that from α = 0.5, the standard error associated is very high, which makes it impossible to affirm that the values are constant. Usually, *E_a_* and *A* change simultaneously with the conversion degree.

**Figure 8:**
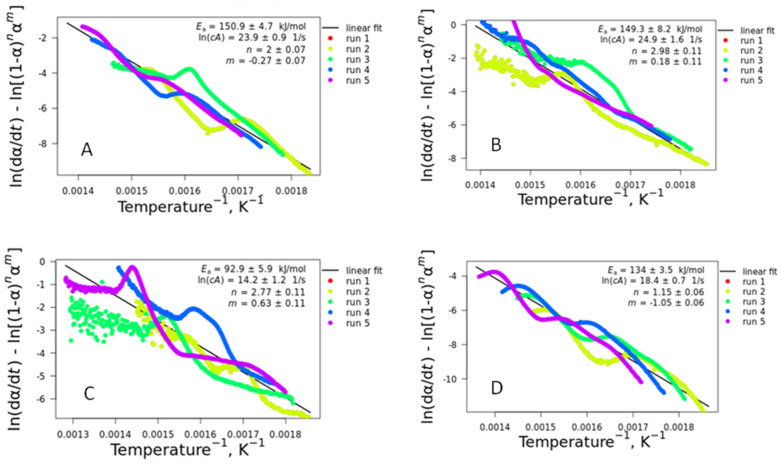
Combined kinetic analysis plots for: (**A**) TPU; (**B**) TPU10%; (**C**) TPU20%; and (**D**) PL 8500 A-10%. The same behavior was observed for runs 1 and 2, hence the curves are overlapped.

Figure 8 shows the combined kinetic analysis that simultaneously uses all heating rates to determine the activation energy, preexponential factor, and the variables *n* and *m*.

When simultaneously analyzed, the different heating rates of the TG curves can give us an insight into the correct *f*(R) function and the resulting *f*(T). It is noteworthy to mention that most of the functions are based on solid-state processes with well-defined functions based on real models [37]. The principle is based on the fact that only the true kinetic model simultaneously fits all experimental data, yielding a unique *f*(T) function. However, in real systems, deviations are expected due to differences in the particle size, particle distribution, changes in interface morphology, and other factors that affect the heat transfer and other characteristics of the thermal behavior.

In general, it is observed that *E_a_* seems to have an interdependence with ln (*cA*), i.e., higher *E_a_* leads to higher *cA*, as has already been reported in the literature [38]. The incorporation of barium sulfate on TPU kept the activation energy and preexponential factor values but changed the *n* and *m* parameters. With further incorporation of the barium sulfate (TPU20%), *E_a_* and *cA* decreased, with distinct *n* and *m* parameters. Finally, lower kinetic parameters were observed for PL 8500 A-10% compared to TPU10%, also with distinct *n* and *m* values. The *n* and *m* values are distinct from the idealized reduced logistic Sestak–Berggren (SB) equation [20]. This is explained by the high standard error in the activation energy values and preexponential factor, mainly in the α = 0.5, where two distinct peaks are observed for all samples analyzed. In spite of the linearity of the activation energy with the conversion degree observed in Figure 7, the behavior seems to not be explained by a single mechanism, at least for the combined analysis. The parameters *m* and *n* describe the shape of the TA curve [39,40]. If the *m* and *n* values follow the theoretical values, it is possible to extract the value of *c* from ln (*cA*) and accurately determine the preexponential factor. If the values do not match, as in our case, this separation cannot be accomplished. Even in this case, these values can be found to describe the effect of the extent of conversion on the reaction rate [20]. The SB equation provides a purely formal description of the kinetics, and it should be applied and understood in this way [41].

**Figure 9:**
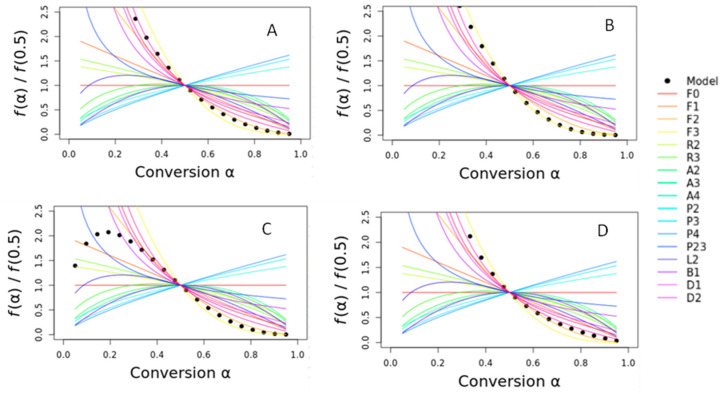
Master plots for (**A**) TPU, (**B**) TPU10%, (**C**) TPU20%, and (**D**) PL 8500 A-10%.

Figure 9A–D shows the following master plots for TPU and the nanocomposites, using the different theoretical models available (the circles represent the experimental fit, while the colored lines represent the following theoretical reaction mechanisms): F0: zero-order reaction; F1: first-order reaction; F2: second-order reaction; F3: third-order reaction. R2: contracting cylinder, R3: contracting sphere, A2, A3, A4—Kolmogorov–Johnson–Mehl–Avrami nucleation growth, P2, P3, P4, P23—power law, B1: classical Prout–Tompkins, D1: one-dimensional diffusion; D2: two-dimensional diffusion; D3: 3D Jander diffusion; D4: 3D Ginstling–Brounshein diffusion; L2: polymer random scission.

Figure 9A,B,D shows some possible reaction mechanisms (F3, D3, and D4) for all α, while for TPU20%, the curve format was similar to L2, B1, A2, A3, and A4-type reaction mechanisms from α = 0.0 to 0.5, and F3, D3, and D4 reaction mechanisms from α = 0.5 to 1.0. Considering the results, it can be concluded that barium sulfate concentrations above 10% in weight change the reaction mechanism, mainly at the beginning of the reaction. It is known that nanoparticles can alter the kinetic parameters of a polymer [42,43]. It is intuitive to assume that nanoparticles can act as some type of concentrator (nucleation, degradation), increase the free volume of the polymer, and facilitate the flow under process, besides altering some mechanical properties, positively influencing the biocompatibility or cytotoxicity of the polymer, and so on. It is noteworthy to mention that some properties can be drastically altered while others are not [44,45,46]. Unfortunately, since the most probable reaction mechanisms are based on the previously kinetic parameters (*E_a_* and *A*), the low R2 in the α ~ 0.5 is indicative that it is very difficult to assume the exact reaction mechanism. However, it can be assumed that the autocatalytic process rules the TPU20%, while diffusion and nucleation/diffusion rule TPU, TPU10%, and PL 8500 A-10%. Our explanation is that, phenomenologically, for all samples, with the exception of TPU20%, the nanoparticle has a small influence, or none, on the heat transfer mechanism, i.e., at the beginning, the heat transfer mechanism seems to be governed by the decelerating process (models of the decelerating type represent processes in which the rate has a maximum at the beginning of the process and decreases continuously as the extent of the conversion increases), while for TPU20%, the sigmoidal (autocatalytic) model seems to rule [20,47]. In the case of 20 wt.% barium sulfate, the particles can help to continuously increase the rate, continuously increasing the extent of conversion, which reaches its maximum at the end of the process. Of course, a mixture of several reaction mechanisms is theoretically expected for this complex polymer (polyurethane), per se, and the nanocomposites. It must be considered that several mechanisms can be simultaneously acting together, which can lead to incongruencies in the results.

### 3.3. Rheology

The rheological behavior of the TPUs was studied using the melt flow index (MFI) and oscillatory rheometer (plate–plate). The results of the MFI at 200 °C were 17.16 g·min^−1^, 23.23 g·min^−1^, and 11.82 g·min^−1^ for TPU10%, TPU20%, and PL 8500 A-10%, respectively. The MFI was 3.66 g·min^−1^ for TPU at 205 °C (at 200 °C, TPU did not flow in the capillary). This means that the incorporation of barium sulfate considerably increased the fluidity of the material. For industrial purposes, this result is extremely interesting because it demands less energy and time. If the cost of the neat polymer is very high, the incorporation of the barium sulfate can considerably decrease the cost of the final product.

**Figure 10:**
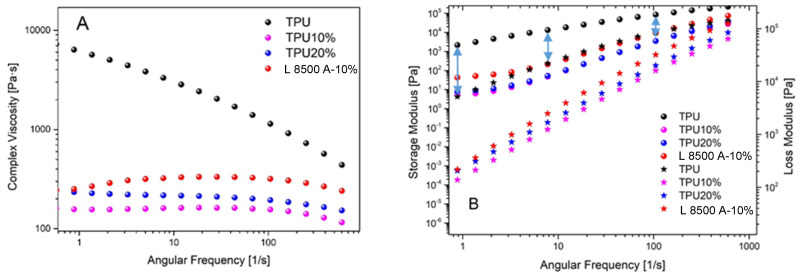
(**A**) Complex viscosity; and (**B**) storage and loss moduli of TPU, TPU10%, TPU20% and PL 8500 A-10%. The arrows are visual representations of the differences between the storage and loss moduli.

Oscillatory rheology was performed, aiming to study the flow under different angular frequencies. At lower angular frequencies, the material has a liquid-like behavior, while at higher frequencies, the predominant behavior is solid-like [48,49]. Figure 10A,B shows the complex viscosity and the storage and loss moduli as a function of the angular frequency for TPU and the nanocomposites, respectively. The magnitude of complex viscosity (total resistance of the flow) of filled TPU is about 1.5 times smaller compared to TPU at the first angular frequencies studied, and the gap among the samples decreases as the frequency increases. Additionally, the complex viscosity decrease for TPU is more attenuated compared to the filled TPUs, indicating that the incorporation of barium sulfate promotes a more linear flow behavior in all frequencies studied. While some particles, such as graphene, can help to improve the hydrogen interaction between the polymeric chains [50], barium sulfate can increase the free volume among the polymeric chains and facilitate the flow among them. This result is particularly interesting in the extrusion process, where more flow stability is indicative of a more controlled process.

The storage modulus measures the elasticity of the material under the frequencies studied. At lower frequencies, the capability to store energy is higher compared to higher frequencies. In this condition, the liquid-like response is predominant. As the angular frequency increases, the difference between the moduli decreases, indicating that the material viscoelastic response predominates at higher angular frequencies, i.e., not a predominance of an elastic or a viscous behavior. The differences between both moduli (when compared to the same TPU) are smaller as the barium sulfate is incorporated in TPU. G’ represents the elastic portion of the viscoelastic behavior, i.e., the higher the molecular restriction of the polymeric chains, the higher the G’ obtained. Hence, it is clear that as barium sulfate is incorporated in TPU, a lower amount of energy is absorbed (as heat) to promote similar deformation when compared to the same angular frequency. For TPU20%, the behavior was very similar to TPU10%, while for PL 8500 A-10%, a higher value was obtained compared to TPU10% and TPU20%, which is indicative of a rigid material in a flow state. For all samples studied, the crossover point (gel point) was not observed (G’=G”), but in the case of PL 8500 A-10%, this point was almost achieved, which means that at the higher frequency studied, the transition from liquid-like to solid-like behavior during gelation was almost achieved.

### 3.4. X-Radiography

**Figure 11:**
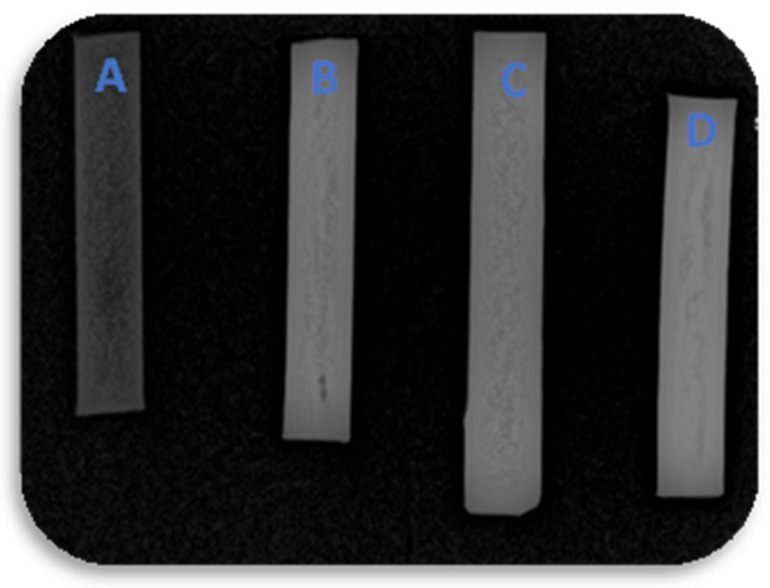
Radiopacity of the TPU and their nanocomposites: (**A**) TPU; (**B**) TPU10%; (**C**) PL 8500 A-10%; and (**D**) TPU20%.

The radiopacity of the TPU and their nanocomposites are presented in Figure 11. The samples were injection-molded, aiming to standardize the width and thickness of the TPUs.

Compared to the TPU sample, all nanocomposites presented higher radiopacity with the incorporation of the barium sulfate, which was expected due to the intrinsic radiopacity of the nanoparticle. In addition, the radiopacity of the TPU10% is very similar to that of the PL 8500 A-10% (commercial grade), indicating that the method proposed was efficient at obtaining similar results.

### 3.5. X-Ray Microtomography

**Figure 12:**
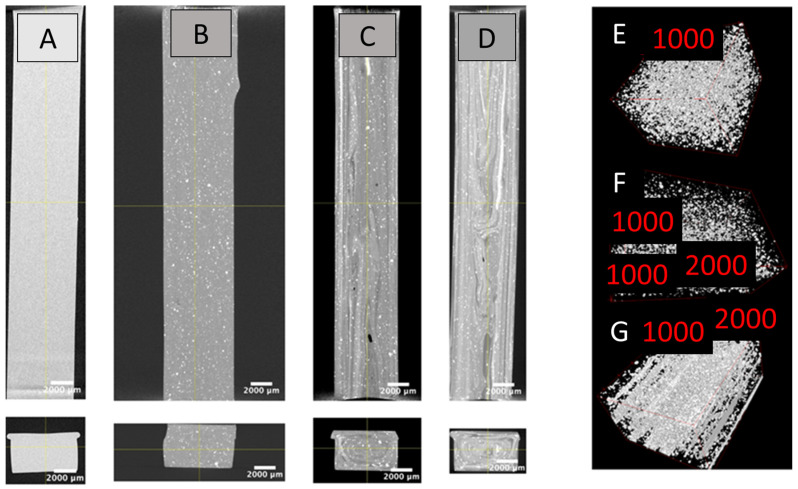
X-ray microtomography of a longitudinal (**top**) and transverse (**bottom**) section of the: (**A**) bare polymer TPU; (**B**) PL 8500 A-10%; (**C**) TPU10%; and (**D**) TPU20%. 3D renderings of the distributions for: (**E**) PL 8500 A-10%; (**F**) TPU10%; and (**G**) TPU20% reveal injection streamlines, dispersion disparities and flow aggregation.

The microstructure of the various nanocomposites was assessed with X-ray microtomography in order to assess the barium sulfate particle volume, surface, and dispersion (Figure 12). Because of the resolution of the scans, the particle analysis threshold was about 20 μm. This means that isolated particles below this size were either counted as matrixes or as bigger particles, provided that volumes with similar attenuation coefficients existed in their vicinity. While the PL 8500 A-10% sample had a relatively heterogenous structure with well-individualized microparticles, the two TPU samples exhibited a coexistence of large, individualized particles and elongated zones with concentrated particles. The elongation of these subregions was seemingly due to higher shears near the injection walls, leading to larger aggregates breaking down into sub-resolution concentrated regions.

**Figure 13:**
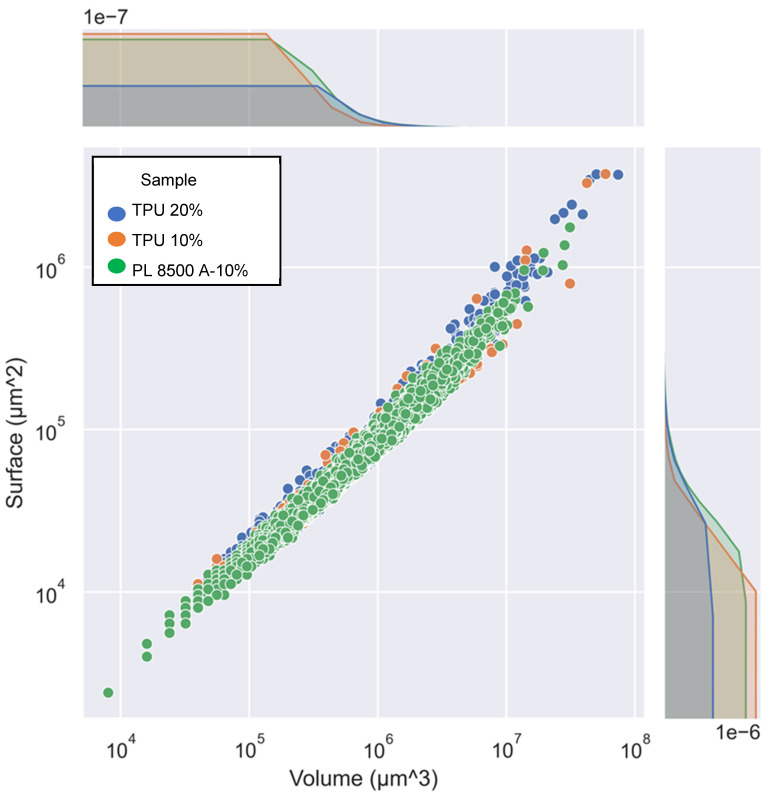
Volume–surface distributions of the three types of aggregates.

Particle analysis led to the conclusion (Figure 13) that the PL 8500 A-10% particles were generally more spherical (for a given volume, their surfaces were smaller) than their counterparts. Similarly, at the high end of the analysis, it is obvious that large fractions in the form of prolate volumes could be attained with the TPU particles, due to extensive shear smearing of particle-rich regions.

## 4. Conclusions

In the present study, two distinct barium sulfate contents (10 wt.% and 20 wt.%) (named TPU10% and TPU20%, respectively) were incorporated into a biomedical-grade polyurethane using a masterbatch procedure. The results were compared with a commercially available (PL 8500 A-10%) radiopaque polyurethane containing 10 wt.% barium sulfate. The structure versus property relationship was discussed in terms of thermal, chemical, physical, rheological, and structural properties. Briefly, the polyurethanes (ether-based) obtained by the masterbatch procedure showed similar properties with the commercially available (ester-based) one. FTIR spectra showed similar absorption bands for all radiopaque polyurethanes when compared to the pristine TPU, indicating that the obtaining procedure did not damage the chemical structure of the polymer matrix. The thermal analyses were evaluated by DSC and TGA. PL 8500 A-10% polyurethane showed a predominant amorphous structure indicated by no presence of any thermal event, while the incorporation of barium sulfate for TPU10% and TPU20% presented well-defined peaks. The incorporation of barium sulfate led to the appearance of a thermal event at approximately 37 °C in the second heating run. In relation to TGA curves, significant differences were noted in the thermal degradation kinetics for the TPU20%, indicating that the polyurethane matrices and the barium sulfate type did not influence this property. The rheological test showed a decrease in the storage modulus and complex viscosity for all nanocomposites compared to the pristine TPU. Differences among the nanocomposites were not significant for this analysis. The radiopacity of the nanocomposites increased with the barium sulfate incorporation, while the X-ray microtomography showed a more spherical particle characteristic for PL 8500 A-10% compared to the other nanocomposites, which showed a large individual particle and an elongated zone with concentrated particles. Hence, the characteristics of the particles (such as agglomeration, format, etc.) did not significantly influence the radiopacity in the same manner as the barium sulfate concentration.

## Data Availability

The data presented in this study are available on request from the corresponding author due to (specify the reason for the restriction).

## Supplementary Materials

The following supporting information can be downloaded at: https://www.mdpi.com/article/10.3390/polym16213086/s1.
