# Supplementary material for: Radiopaque Polyurethanes Containing Barium Sulfate: A Survey on Thermal, Rheological, Physical, and Structural Properties
Source: Polymers (Basel). 2024 Oct 31;16(21):3086. doi: 10.3390/polym16213086 (PMC11548690; doi:10.3390/polym16213086)
Supplement: Supplementary file 1 [file polymers-16-03086-s001.zip › polymers-3289598-Supplementary Material.pdf]

Supplementary Material S1

The thermal decomposition of most polymers is an endothermic process, and it is accompanied by a mass loss (due to low molecular weight species or gas, for example) and changes in the heat transfer phenomena. These changes are recorded by TGA as a function of time or temperature and can be converted to the degree of conversion (α) according to Equation 1:

$$\alpha = \frac{m_t - m_0}{m_f - m_0} = \frac{\Delta m_t}{\Delta m_{total}} \quad (1)$$

where α is the conversion degree, m_t is the sample mass at time t, m_0 is the initial sample mass and m_f is the final sample mass.

Higher the heating rate (β), larger the temperature gradient in the sample, and the excessively fast reaction rate causes considerable self-cooling or self-heating effect due to the enthalpy of the reaction. To avoid such complications caused by these heat transfer phenomena as the mass changes, proper heating rates may be used (preferentially lower heating rates).

The thermal degradation kinetic behavior was studied using the isoconversional differential Friedman method (Equation 2) and the advanced integral Vyazovkin method (Equations 3 and 4)

$$ln\, ln \left(\frac{d\alpha}{dt}\right)_{\alpha,i} = ln\,[f(\alpha)A_\alpha] - \frac{E_a}{RT_{\alpha,i}} \quad (2)$$

where α is the conversion degree, t is the time, A_α is the frequency factor (pre-exponential), E_a is the activation energy, $T_{\alpha,i}$ is the absolute temperature, R is the universal gas constant, and $f(\alpha)$ is the differential conversion temperature.

$$\Phi(E_\alpha) = \sum_{i=1}^{n} \sum_{j\neq 1}^{n} \frac{J[E_\alpha, T_i(t_\alpha)]}{J[E_\alpha, T_j(t_\alpha)]} \quad (3)$$

$$J(E_\alpha, T(t_\alpha)) = \int_{t_\alpha - \Delta\alpha}^{t_\alpha} A\, exp\left[\frac{-E_a}{RT(t)}\right] dt \quad (4)$$

Kissinger' (eq. 5) consider only the peak temperature at α = 0.5, not considering the whole conversion degree with temperature.

$$ln\left(\frac{\beta}{T_p^2}\right) = ln\left(\frac{AR}{E}\, f'(\alpha_p)\right) - \frac{E}{RT_p} \qquad (5)$$

The combined kinetic analysis (eq. 6) estimates the activation energy, preexponential factor and the most probable reaction mechanism (by determining the *n* and *m* and comparing them with the values of the theoretical models in the solid-state reaction):

$$ln\left(\frac{d\alpha}{dt}\right) - ln\, ln\,[(1-\alpha)^n \alpha^m] \; = ln(cA)\frac{E_a}{RT} \qquad (6)$$

where *n* and *m* are variables which values are indicative of the most probable reaction model.
